# Supplementary material for: Synthesized spatiotemporal mode-locking and photonic flywheel in multimode mesoresonators
Source: Nat Commun. 2022 Oct 27;13:6395. doi: 10.1038/s41467-022-34103-0 (PMC9613675; doi:10.1038/s41467-022-34103-0)
Supplement: Supplementary file 1 — Supplementary Information [file 41467_2022_34103_MOESM1_ESM.pdf]

Supplementary Information for “Synthesized spatiotemporal mode-locking and photonic flywheel in multimode mesoresonators”

Mingming Nie[1, *], Kunpeng Jia[2, *], Yijun Xie[1], Shining Zhu[2], Zhenda Xie[2, *], and Shu-Wei Huang[1, *]

[1]*Department of Electrical, Computer and Energy Engineering, University of Colorado Boulder, Boulder, Colorado 80309, USA*
[2]*National Laboratory of Solid State Microstructures, School of Electronic Science and Engineering, College of Engineering and Applied Sciences, School of Physics, and Collaborative Innovation Center of Advanced Microstructures, Nanjing University, Nanjing 210093, China*
[*]*Corresponding author: mingming.nie@colorado.edu, jiakunpeng@nju.edu.cn, xiezhenda@nju.edu.cn, shuwei.huang@colorado.edu*

This Supplementary Information for “Synthesized spatiotemporal mode-locking and photonic flywheel in multimode mesoresonators” provides additional information for the main text.

- In Section I, we summarize the advantages of GRIN-MMF FP mesoresonators.
- In Section II, we provide the information of Q factors for other spatial modes, the simulated mode profiles and group velocities for the simulated modes.
- In Section III, we provide the other comb states in the experiment.
- In Section IV, we provide the other experimental details.
- In Section V, we demonstrate soliton generation in another two different GRIN-MMF FP mesoresonators with different FSRs compared with the one in the main text. Solitons are generated by different acoustic modes or different optical modes.
- In Section VI, we provide the detailed experimental information for phase locking the SBS frequency shift to a reference RF signal.
- In Section VII, we present detailed numerical results for STML DKS.
- In Section VIII, we measure the noise floor of the frequency discriminator.
- In Section IX, we provide the detailed information of the timing jitter measurement setup.
- In Section X, we present detailed noise analysis for the SBL soliton.

I. SUMMARY OF ADVANTAGES OF GRIN-MMF FP MESORESONATORS

Higher quality factor is just one of the many benefits we gain from GRIN-MMF FP mesoresonator platform compared to the HNLF FP mesoresonator in our previous work. Figure S1 summarizes the advantages of the GRIN-MMF FP mesoresonator that lead to enhanced photonic flywheel performances and the STML DKS demonstration in this paper. In addition to higher quality factor, the low modal dispersion unique to the GRIN-MMF renders the STML DKS possible as such low modal dispersion can be effectively compensated by the cross-phase modulation (XPM) between different spatial modes. Furthermore, the GRIN-MMF provides a large number of acoustic modes that can be phase matched for stimulated Brillouin scattering (SBS) and thus it greatly relaxes the fabrication tolerance of the mesoresonator length for finding the suitable pump-SBL mode pair to apply the two-step pumping scheme. Finally, the large mode volume and low Kerr nonlinearity result in low thermal and quantum noises that are also responsible for the narrower comb linewidth and lower timing jitter demonstrated in this paper. In contrast, our previous photonic flywheel work with HNLF-SMF is limit by the quantum noise.

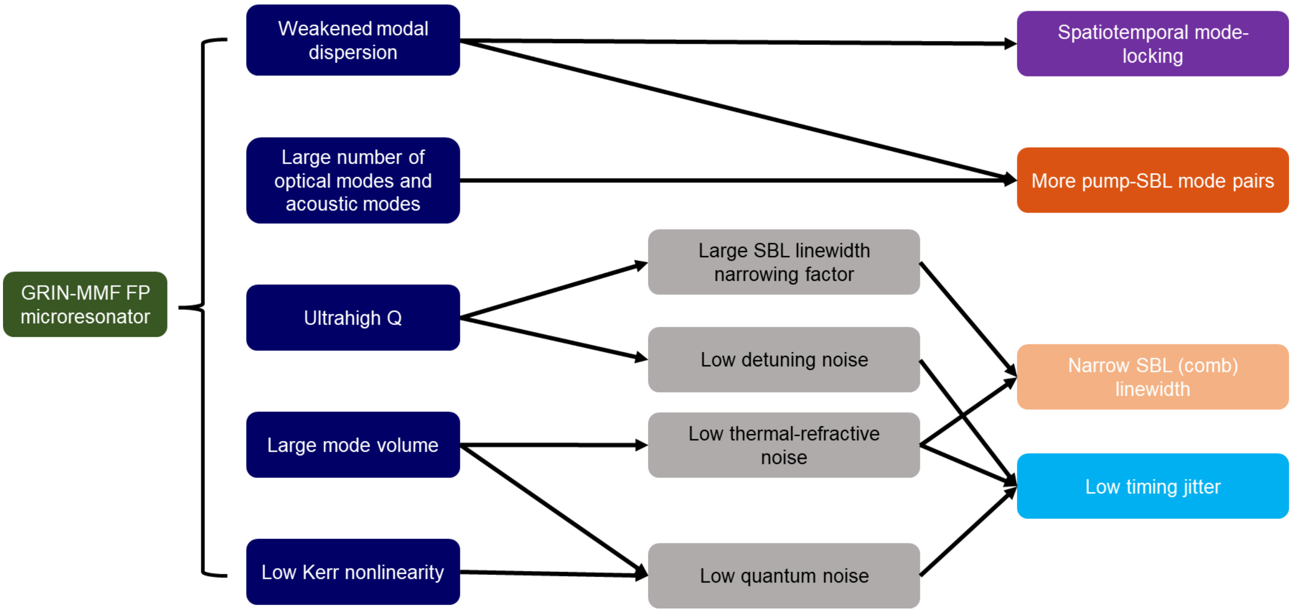

Fig. S1. Summary of advantages of GRIN-MMF FP mesoresonators.

II. MODE ANALYSIS OF GRADED-INDEX FIBER

We also measure the Q factors for the other three modes in Fig. 2b in the main text. As shown in Fig. S2, the Q factors are larger than 310 million (M) for these modes, verifying the large mode area induced low diffraction loss.

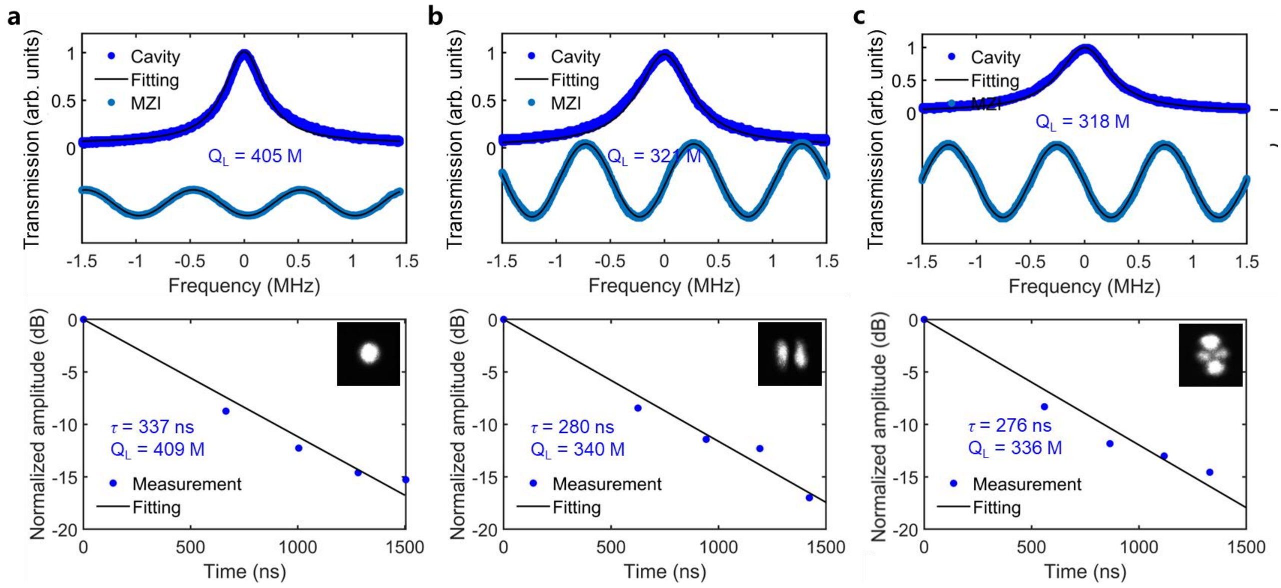

Fig. S2. Q factor measurement for the other three modes (mode profiles in the inset). Top row: transmission spectrum of the ultrahigh-Q mode and MZI (blue lines) at 1550 nm. Bottom row: ring-down traces. ("M" represents "million")

We numerical calculated the linearly polarized (LP) mode through the code in Ref. [1]. In fact, there are hundreds of modes in GRIN-MMFs and we only show the first ten modes in Fig. S3, which are classified into four groups in the order of effective refractive index. The experimental mode profiles in Fig. 2b can be viewed as superpositions of different LP eigenmodes, especially for the second mode and fifth mode. The pump mode (SBL mode) has profiles similar to the LP_{11} (LP_{21}) mode, but in fact they are not 100% identical.

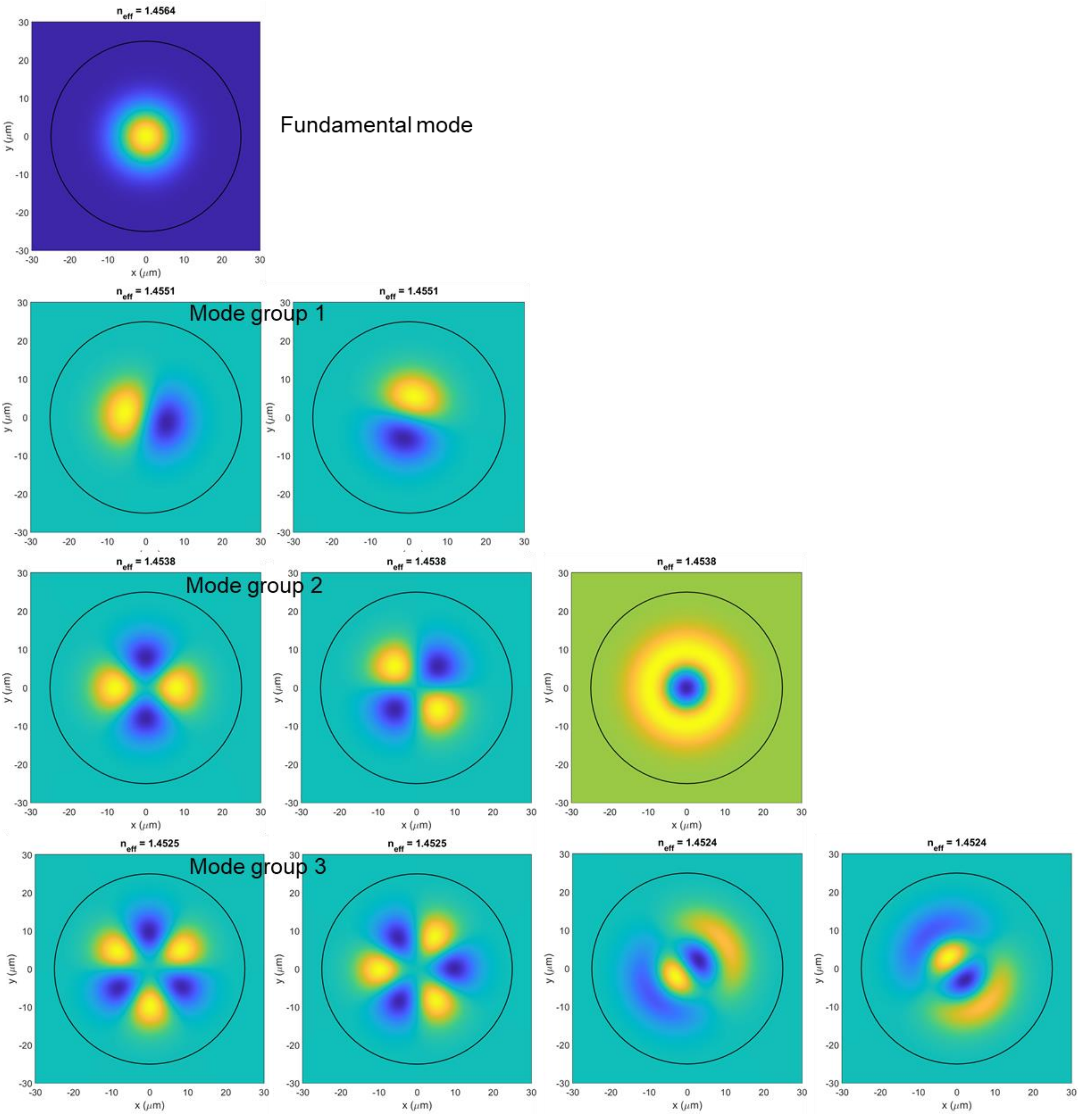

Fig. S3. LP eigenmodes of GRIN-MMFs. The black circular lines indicate the core edge of GRIN-MMF.

The simulated GVD for the ten modes in Fig. S3 is shown below in Fig. S4, indicating four mode groups. The simulated GVD is around -28 fs^2/mm for all the ten modes.

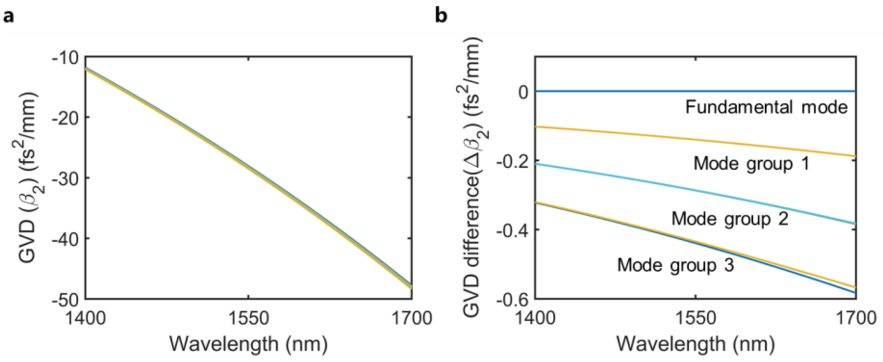

Fig. S4. Simulated GVD. (a) and GVD difference (b) with respect to the fundamental mode for the modes in Fig. S3.

III. OTHER COMB STATES

We obtain other comb states for pump soliton and SBL soliton, as shown in Fig. S5.

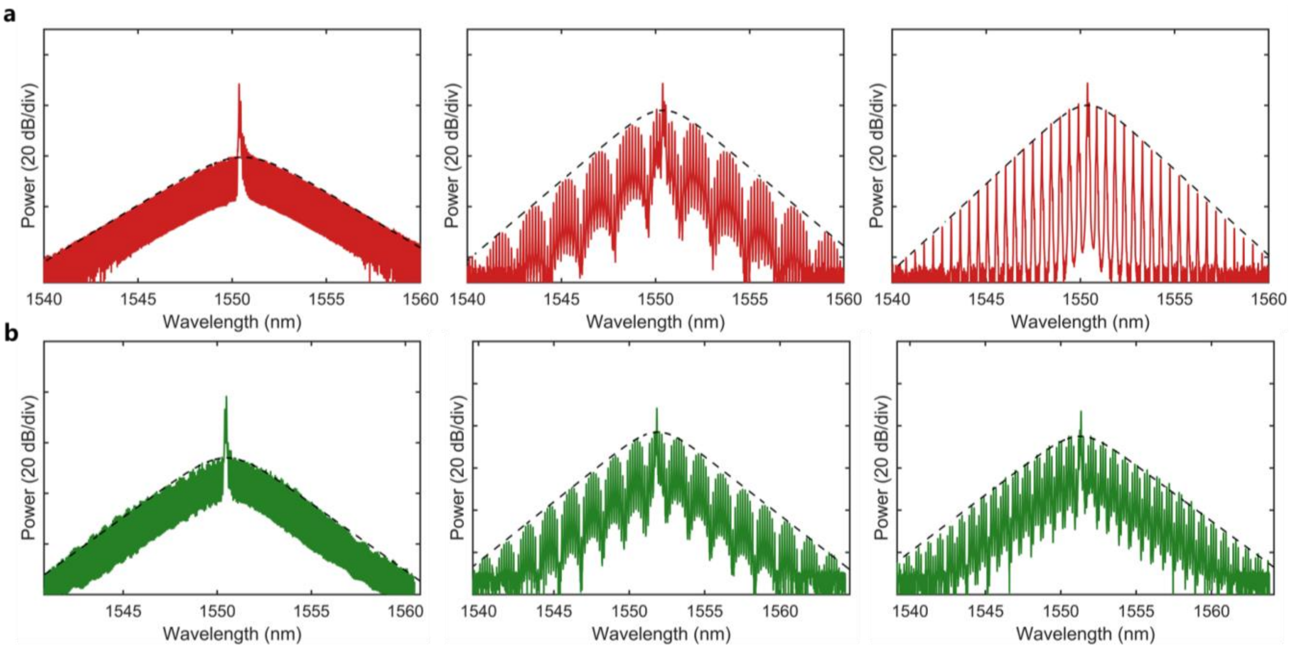

Fig. S5. Other comb states for pump soliton (a) and SBL soliton (b). Dashed lines represent the fitted spectral envelope.

IV. EXPERIMENTAL DETAILS

As shown in Fig. S6, in the mesoresonator mount, two pairs of screw/spring sets are used to impose stress on the FP mesoresonator to tune the SBS frequency shift and selectively excite soliton microcombs among the pump soliton, the SBL soliton and STML soliton.

The coupling loss from single-mode fiber to the multimode mesoresonator is about -5 dB, mainly due to the mode mismatching.

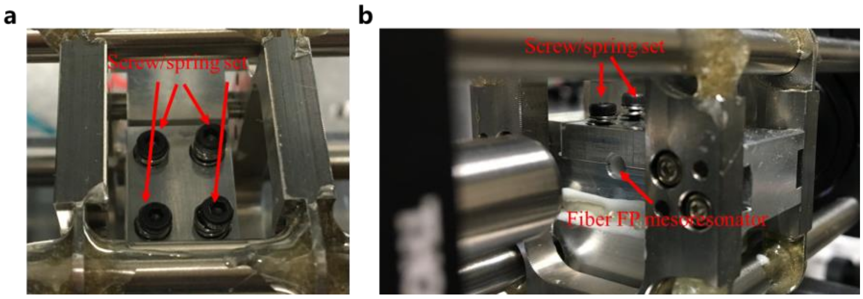

Fig. S6. Stress tuning using screw/spring set. (a) top view; (b) side view.

V. SOLITONS WITH OTHER GRIN-MMF FP MESORESONATORS

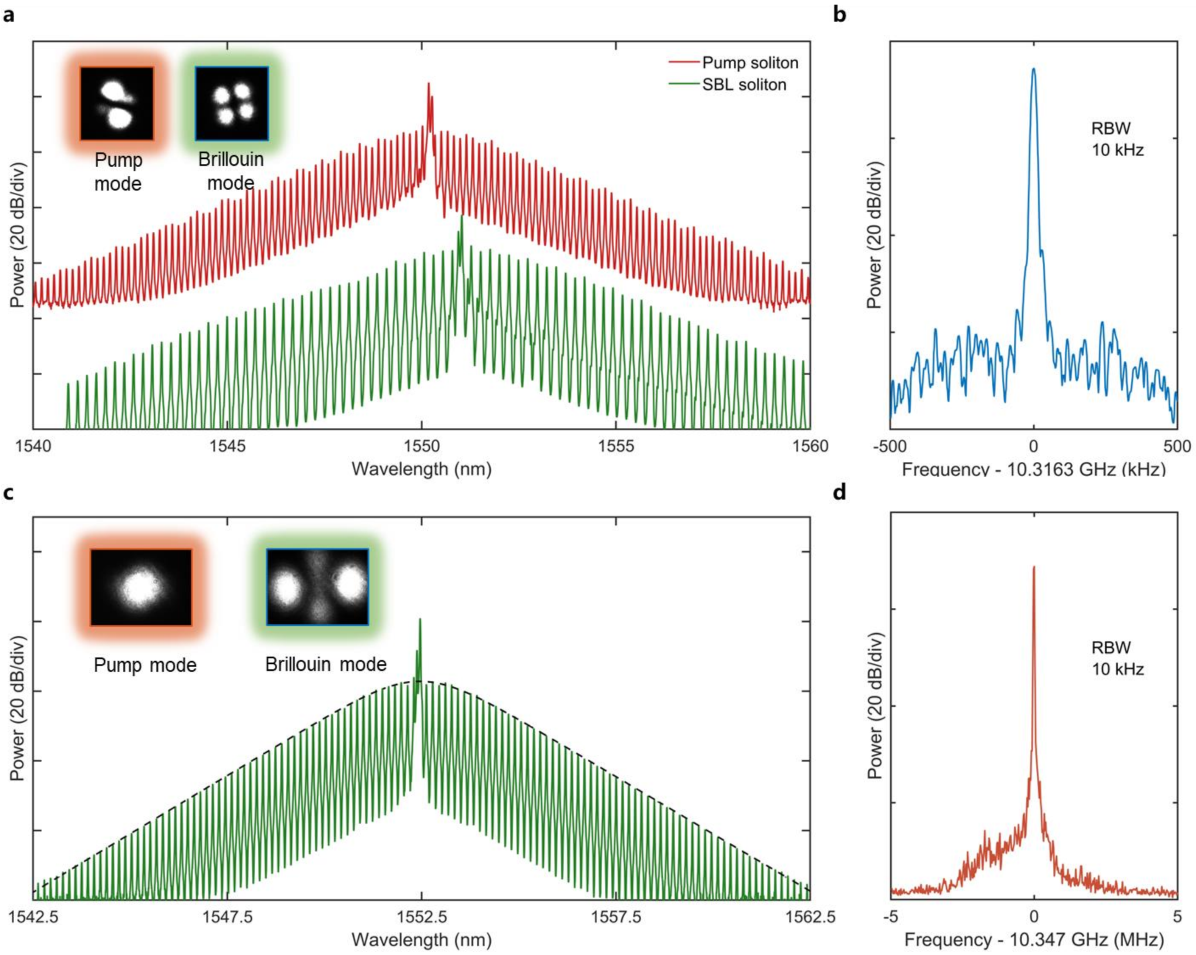

Fig. S7. (a)(b) Pump soliton and SBL soliton generated in a second GRIN-MMF FP mesoresonator with FSR of 10.087 GHz. (c)(d) SBL soliton generated in a third GRIN-MMF FP mesoresonator with FSR of 10.086 GHz. (a)(c) spectra; (b)(d) Measured SBS frequency shift when SBL soliton is generated.

We also realize the selective excitation of solitons (Fig. S7a) in a second high-Q GRIN-MMF FP mesoresonator with FSR of 10.087 GHz, which is 8 MHz larger than that the one in the main text. Despite the same mode profiles (pump mode and SBL mode) recorded by the camera, we find the SBS frequency shift of 10.3163 GHz (Fig. S7b) is smaller than the measured value of 10.343 GHz in the main text, owing to the SBS process through another acoustic mode. Interestingly, the simulation [2] shows the spectral distance between adjacent acoustic resonances is ~27 MHz, which is perfectly with our experimental results.

Besides pump-SBL mode pairs with different acoustic modes, we also achieve SBL soliton generation from pump-SBL mode pairs with different optical mode profiles (Fig. S7c and S7d), in a third high-Q GRIN-MMF FP mesoresonator with FSR of 10.086 GHz, which is 7 MHz larger than that the one in Fig. 2.

With mitigated GVM and broadened intermodal SBS gain spectrum between a great variety of high-Q spatial modes and different acoustic modes, it is easy to find pump-SBL mode pairs with high-Qs and apply the scheme presented here for feedback-free soliton combs generation, which will definitely lower the fabrication requirement for cavity length.

VI. PHASE LOCKING LOOP

Coherence between the SBL and the pump is the prerequisite of a successful STML DKS demonstration. With the two-step pumping scheme, the pump DKS and the SBL DKS are intrinsically coherent to each other. Long-term coherence of STML DKS can also be guaranteed with an active stabilization using a home-built optical phase lock loop (PLL) as shown in Fig. S8. The 10.343-GHz beat signal between the pump and SBL is first divided by 8 times and then compared with a good RF reference signal (HP, E4431B ESG-D) by using a double sideband mixer. The error signal from the mixer is sent to a proportional–integral–derivative servo that controls the pump laser frequency through the current driver. The bandwidth of the current driver is 10 kHz.

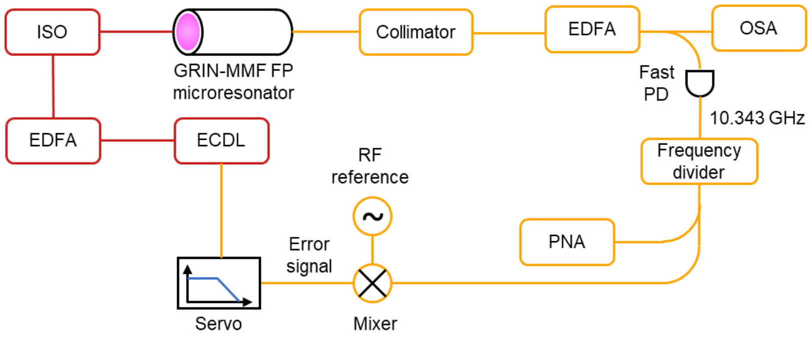

Fig. S8. Schematic of the PLL setup. ECDL: external-cavity diode laser, EDFA: Erbium-doped fiber amplifier, ISO: optical isolator, OSA: optical spectrum analyzer, PD: photodetector, PNA: phase noise analyzer.

VII. EXISTENCE CONDITIONS FOR STML DKS

To achieve the STML DKS, two conditions must be satisfied: (i) good coherence between the different pump lasers for different soliton modes; (ii) small group velocity mismatch (GVM) between the different soliton modes. With the two-step pumping scheme, the pump DKS and the SBL DKS are intrinsically coherent to each other. Long-term coherence can also be guaranteed with an active stabilization as described in Supplementary Information Section VI and Fig. 3d. Thus, the existence of STML DKS in our system only relies on the balance between GVM and XPM that can be studied by numerically solving two coupled LLEs [3] for the DKSs in the two mode families.

Figure S9a plots the existence map of STML DKS as a function of normalized FSR difference resulting from GVM and normalized pump power that controls the magnitude of the XPM effects. The overlap integral between the mode 2 and mode 4 in Fig. S3, representing the pump mode and SBL mode in the experiment, is calculated to be 0.624 [4] and consequently the XPM coefficient is 1.24 times the SPM coefficient. For the existence of STML DKS, the normalized FSR difference should be kept below 10^{-4}, which is granted by the low modal dispersion of the GRIN-MMF. The STML DKS existence range first increases with the pump power, benefiting from the increased nonlinear mode coupling, and then decreases with pump power as the soliton perturbation grows to destabilize the system. Example STML DKS profiles and spectra decomposed into its two eigenmodes are shown in Fig. S9b. Of note, there is a slight offset between the center frequencies of the two constituent DKSs as a result of soliton trapping from the balance between GVM and XPM. Fig. S9c plots the existence map of STML DKS when the XPM coefficient is reduced to half of the SPM coefficient. Lower XPM coefficient can be achieved by either combining modes with less symmetry (i.e. small overlap integral) or orthogonal polarizations. Of note, the tolerance to GVM is greatly enhanced when the XPM coefficient relative to the SPM coefficient is reduced.

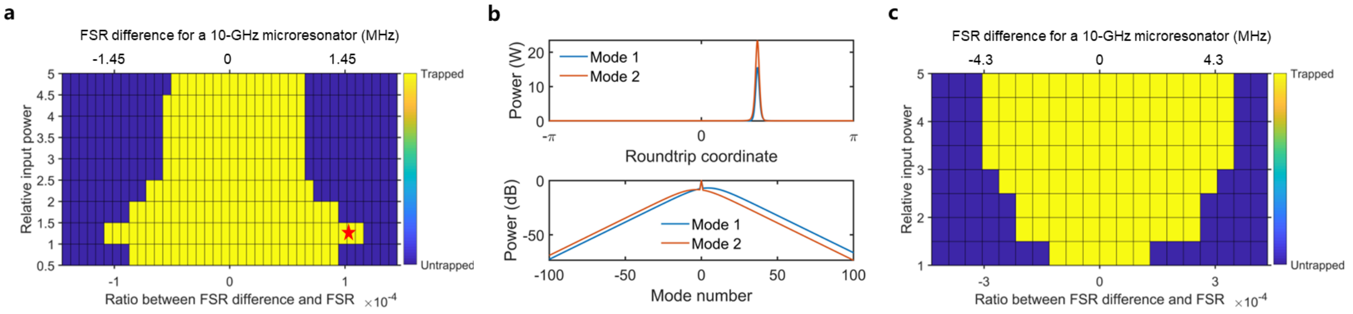

Fig. S9. (a) Existence map of STML DKS as a function of normalized FSR and normalized pump power. The XPM coefficient is 1.24 times the SPM coefficient. The input pump power is normalized to the DKS threshold pump power when XPM is absent. (b) Example STML DKS profiles and spectra decomposed into its two eigenmodes, obtained at the condition labeled by the red star in (a). (c) Existence map of STML DKS as a function of normalized FSR and normalized pump power. The XPM coefficient is lowered to 0.5 times the SPM coefficient.

VIII. LINEWIDTH MEASUREMENT

Our frequency discriminator is first tested by a known reference laser from NIST at Boulder, as shown in Fig. S10. The measured fundamental linewidth is 0.3 Hz and verify the reliability of the measured comb linewidths at sub-hertz level. The noise floor tested by our SBL laser indicates the minimum fundamental linewidth we can measure is about 3 mHz, which is limited by both the test laser source [5] and the shot noise of the BPD.

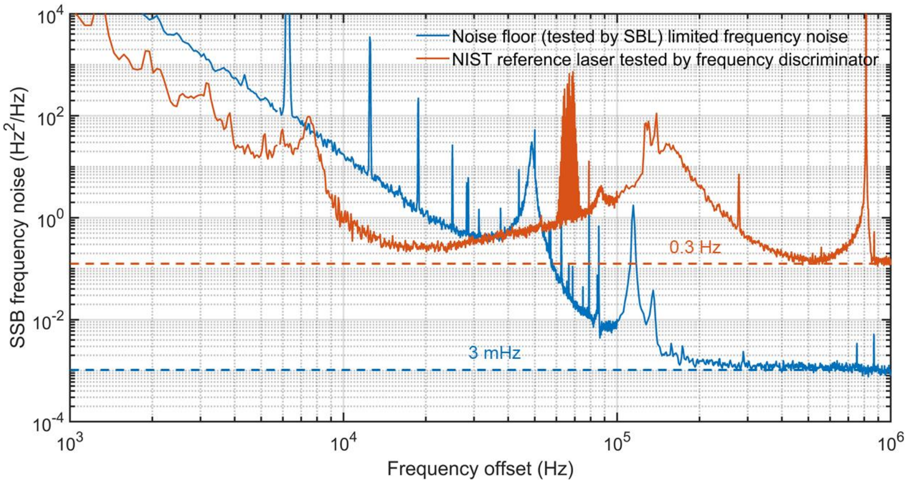

Fig. S10. Optical frequency discriminator characterization with NIST reference laser.

IX. TIMING JITTER MEASUREMENT SETUP

The detailed schematic of ARMI timing jitter measurement setup can be found in Ref. [6]. The method can be summarized briefly below. For each comb line, its optical frequency can be written as $f_k = kf_{rep} + f_{ceo}\left(k = 1, 2, 3...\right)$, where f_{rep} is the repetition rate and f_{ceo} is the carrier-envelope-offset frequency. Two frequency modes (f_m and f_n) are used to extract phase noise $\left(m-n\right) f_{rep}$ by subtracting the noise of common mode f_{ceo}.

In our experiment, two filters (1545 nm with 2-nm FWHM bandwidth and 1555 nm with 2-nm FWHM bandwidth) are employed to filter out the two main frequency modes (f_m and f_n). An unbalanced Michelson interferometer with an 82-m-long fiber delay line (time delay of $\tau = 410$ ns) is used for converting phase noise into intensity noise. The delay line is implemented by using an 80-m-long fiber PZT stretcher and 2-m-long extra fiber. The length of the fiber delay line is chosen to make a compromise between the timing detection sensitivity (-174 dBc/Hz at 10.079 GHz) proportional to τ and measurement bandwidth scaling with $1/\tau$ (2.5 MHz). A delay control unit (DCU) consisting of two electrically-driven delay lines is implemented to compensate the group velocity mismatch between the two frequency modes and maximize the interferometer output voltage. An acousto-

optic frequency shifter (AOFS) with frequency shift of 50 MHz is inserted in the fiber delay line to avoid background noise in the baseband by synchronous detection. The interferometer output then contains the frequency noise of each frequency mode weighted by the delay time at center frequency of $f_{\mathrm{AOM}} = 100$ MHz, in the form of phase noise as $\delta\left[\left(f_m + f_{\mathrm{AOM}}\right)\tau\right]$ and $\delta\left[\left(f_n + f_{\mathrm{AOM}}\right)\tau\right]$. We then filter the photo-detected signal of each mode at $f_{\mathrm{AOM}} = 100$ MHz and mix them by an RF mixer to reject the common-mode f_{ceo} noise. This down-converted RF mixer output contains the repetition rate frequency noise in the form of phase noise (timing jitter) as $\delta\left[(m-n) f_{rep}\tau\right]$.

During the measurement, a delay-locked loop (DLL) is introduced for locking the delay to the comb under test and preventing optical frequency drift. Served as error signal, the mixer output is fed to the servo, whose output is applied to the PZT stretcher in the fiber delay line. It is important to keep the locking bandwidth as low as possible to ensure broadband characterization because the free-running comb jitter can be analyzed only outside the DLL locking bandwidth. In this work, we used a 100 Hz locking bandwidth. As a result, the jitter PSD from 100 Hz to 2.5 MHz can be measured. The measured voltage PSD (intensity noise) is converted to the frequency noise by the transfer function $T(f)$ resulting from the fiber delay line [6–8]

$$T(f) = \mathrm{V}_p \frac{\left|1 - e^{-i2\pi f\tau}\right|}{\left|i \times f\right|} (\mathrm{V/Hz}) \tag{SE 1}$$

where V_p is half of the peak-to-peak voltage from the mixer output (within Fourier frequency range < f_{AOM}). Equivalent timing jitter or repetition rate phase noise can then be converted from the frequency noise. Of note, the transduction from amplitude to timing jitter in the ARMI setup is extremely low. The soliton RIN as well as other common noise for the two filtered frequency modes do not influence the accuracy of the measured soliton jitter [7].

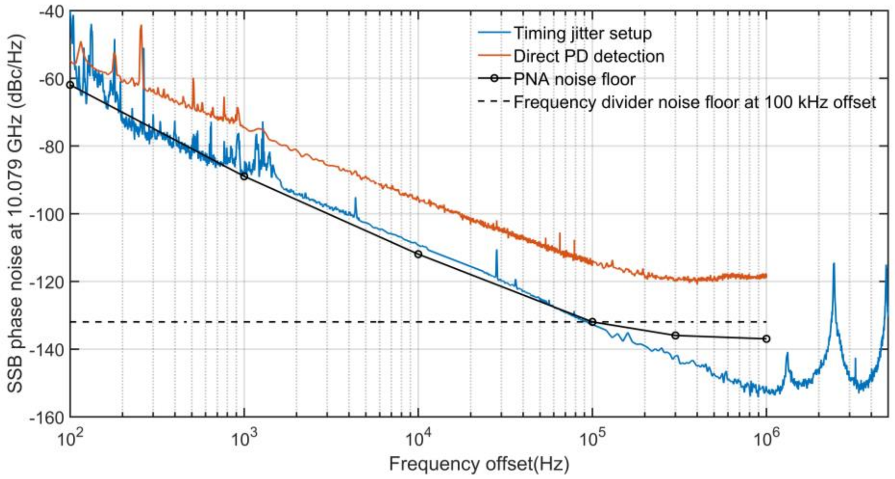

Fig. S11. Comparison of SSB phase noise of locked soliton repetition rate with different methods, the timing jitter setup and the direct PD detection. For the latter method, we inject the comb lines into the fast PD, electrically frequency divide the 10.079 GHz RF signal by 8 times (with a low noise floor) and then analyze the divided signal with a PNA. The noise floor of the frequency divider has been included in the plot of "PNA noise floor".

The timing jitter measurement setup is designed for low repetition rate PN, i. e., small $\delta\left[(m-n) f_{rep}\tau\right]$ thus low peak-to-peak voltage with DLL. Large phase noise will lead to high peak-to-peak voltage with DLL and invalidate the linear approximation around the locking point, which locates at the center of the sinusoidal voltage function. Therefore, the measured voltage PSD is incorrect and smaller than the correct one. We show in Fig. S11 the comparison of measured results for the STML soliton, with both the ARMI timing jitter setup and the conventional direct method using a fast PD and a phase noise analyzer. According to Fig. S11, the result from timing jitter setup is incorrect and smaller than the other one (the correct one) due to the large phase noise.

Apart from the single SBL soliton, we also measure the repetition rate PN of the 2-FSR SBL soliton comb as shown in Fig. S12.

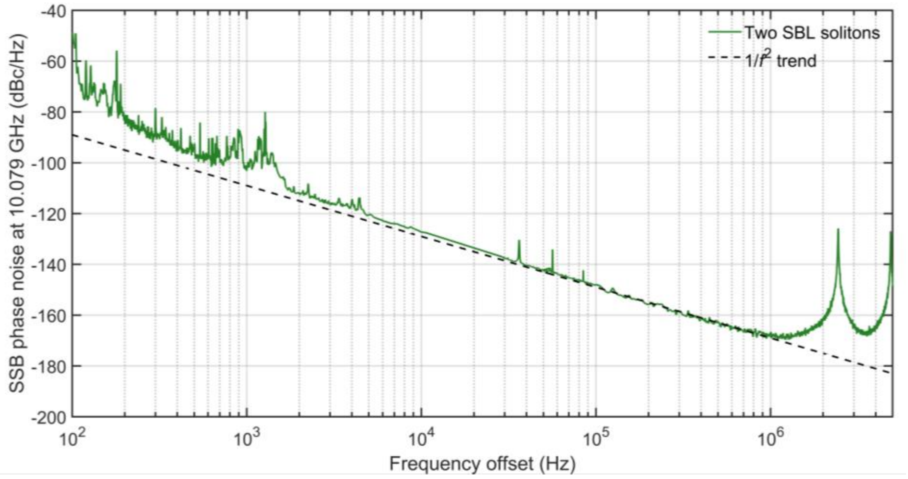

Fig. S12. SSB repetition rate PN of the 2-FSR SBL soliton.

X. SBL SOLITON REPETITION RATE PHASE NOISE ANALYSIS

A. Detuning effect on RIN

We will first study the detuning effect on relative intensity noise (RIN) (intracavity power fluctuations) for the output of our high-Q mesoresonator, which helps to later distinguish the origin of soliton repetition rate phase noise (PN), whether from the SBL RIN or soliton RIN. As shown in Fig. S13, we record the output RIN PSD of two modes (fundamental mode at low input power and pump mode at high input power) by tuning the ECDL into the resonance when SBS is below the threshold. The intracavity RIN is inherently attributed to the frequency noise of the resonant laser. In addition, the peak position on the Fourier frequency axis is related to both the resonance linewidth and laser detuning, given by [9]

$$S_{RIN}(\Omega) \propto \frac{\eta^2 \Delta^2 \kappa^2 \left[(1-\eta)^2 \kappa^2 + \Omega^2\right]}{\left[\Delta^2 + (\kappa/2)^2\right]\left[(\Delta-\Omega)^2 + (\kappa/2)^2\right]\left[(\Delta+\Omega)^2 + (\kappa/2)^2\right]} S_{\phi}(\Omega), \qquad \text{(SE 2)}$$

where Δ is the laser detuning from the cavity resonance, $\kappa/2\pi$ is the cavity FWHM linewidth and Ω is the Fourier frequency. As shown for both modes with different input powers, clear RIN peak shifts with blue-detuning variation. Therefore, the RIN peak is a convenient indicator to monitor the laser detuning variation.

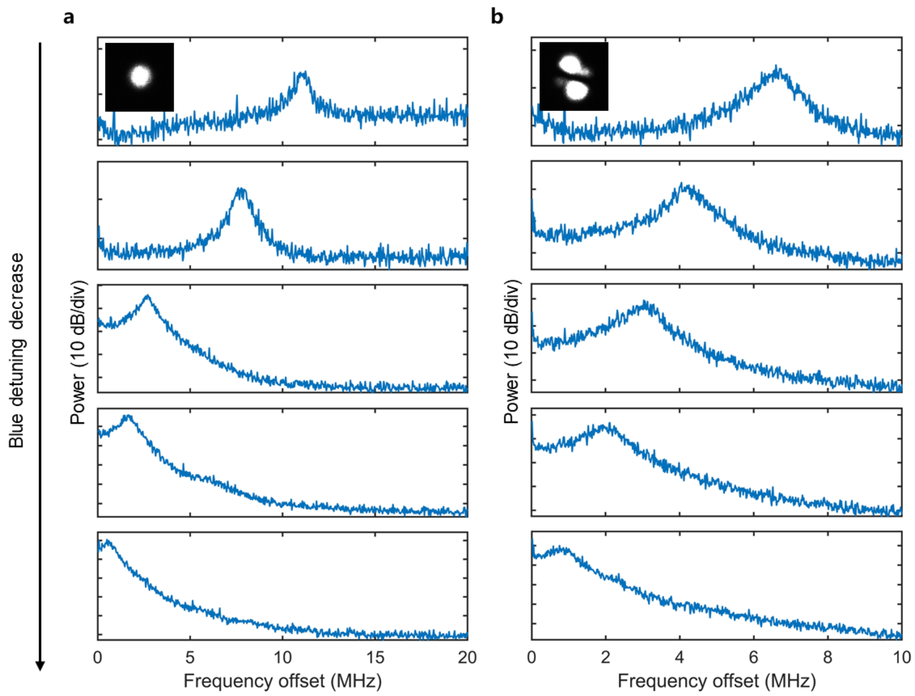

Fig. S13. Detuning effect on relative intensity noise. With decreased blue detuning, the peak of intensity noise shifts towards zero offset frequency for both the fundamental mode at low power (a) and pump mode at high power (b).

B. SBL RIN, soliton RIN and influence on timing jitter

In this section, we precisely measure the RINs of the pump, the SBL before and after SBL soliton generation and the SBL soliton RIN, based on which we determine the dominant factor influencing the SBL soliton repetition rate PN.

First, we measure the total RIN of pump plus generated SBL before SBL soliton generation (Fig. S14) with different SBL intensities. As the intensity of SBL increases, the total RIN decreases, indicating the SBL RIN is smaller than the pump RIN. When the intensity of SBL is 10 dB stronger than that of the pump, the total RIN, which is dominated by the SBL RIN, is ~10dB smaller than the pump RIN before SBL generation. According to Ref. [10], we calculate the transduction coefficient from pump RIN to SBL RIN (inset of Fig. S14, details see Appendix), with transduction coefficient of -10 dB at low offset frequencies. The transduced SBL RIN from pump RIN is also overlaid in Fig. S14, which agrees well with the measured total RIN dominated by SBL RIN. Of note, the deviation at the low offset frequencies is attributed to the technical noise.

By fine tuning the wavelength of ECDL, we obtain the 2-FSR SBL combs in Fig. 2b (main text). In this case, SBL intensity is ~7 dB stronger than pump's intensity. By simultaneously monitoring the spectrum, the total RIN, the soliton RIN, the comb repetition rate PN and the comb fundamental linewidth, we find all the measured results strongly depends on the wavelength of ECDL. As shown in Fig. S15, when we tune the wavelength of ECDL towards red-detuned side, (i) the total RIN decreases by 4 dB (Fig. S15a), (ii) the soliton RIN decreases by 5 dB (Fig. S15b), (iii) comb repetition rate PN decreases by ~5 dB (Fig. S15c) and (iv) the SBL fundamental linewidth narrows from 6.1 Hz to 0.4 Hz thus decreases by 12 dB (Fig. S15d). Of note, the total RIN is measured by filtering the pump, SBL and some comb lines together with a 2-nm bandpass filter, leading to the dip at 1.2 MHz resulting from the interference between pump, SBL and comb lines. In this case, the intensity of SBL is ~7 dB larger than the pump's, which means the total RIN is dominated by SBL RIN. Interestingly, the total RIN peak shifts as shown by the blue arrow in Fig. S15a, indicating the reduced value of red detuning for SBL according to detuning effect discussed above in Section VI A. Therefore, SBL linewidth narrowing in Fig. S15d can be explained due to the smaller detuning induced better phase matching during SBS process [11].

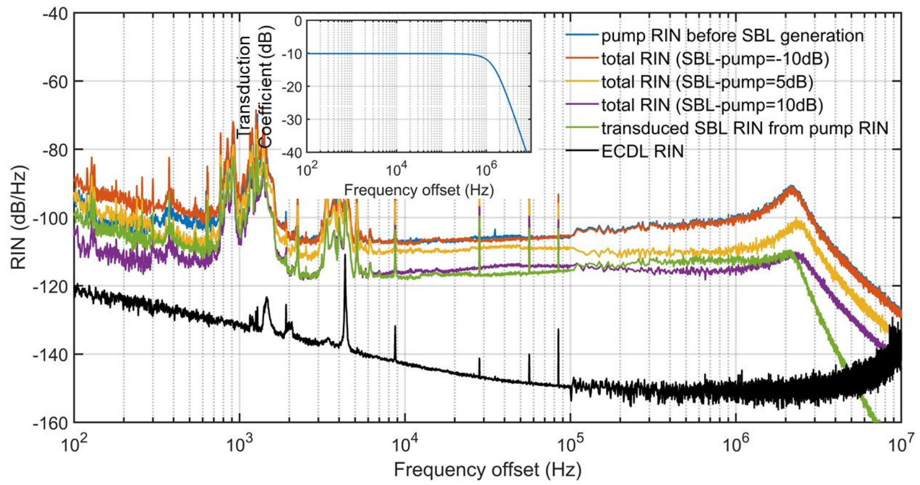

Fig. S14. RIN for pump before SBL generation, after SBL generation with different SBL intensities, transduction from pump, and the ECDL. Inset: transduction coefficient from pump RIN to SBL RIN.

Soliton RIN does not dominate the SBL soliton timing jitter. In Fig. S15b, the intensity of soliton RIN peak at 1.2 MHz sharply decreases by ~25 dB with decreased SBL laser detuning. As for the comb repetition rate PN in Fig. S15c, there is a small peak at 1.2 MHz at large SBL detuning while it disappears at small SBL detuning. We attribute the small peak to the soliton RIN transduced soliton repetition rate PN, indicated by the blue arrows in Fig. S15b and S15c. However, the soliton RIN is not the dominant origin for the soliton PN due to the inconsistent variation between 5 dB (Fig. S15c) for soliton PN and ~25 dB (Fig. S15b) for soliton RIN peak at 1.2 MHz. Furthermore, the relatively small comb bandwidth and thus weak self-steepening effect will ensure small transduction coefficient from soliton RIN to soliton PN.

Detuning noise from SBL does not dominate the SBL soliton timing jitter. When decreasing the SBL detuning, the SBL phase noise at high offset frequencies decreases by 12 dB (Fig. S15d) while the repetition rate PN only decreases by 5 dB (Fig. S15c). The inconsistent intensity changes suggest that the soliton repetition rate PN is not determined by the SBL phase noise.

SBL RIN dominates SBL soliton timing jitter. Considering the change of 5 dB (Fig. S15c). for repetition rate PN during the frequency tuning process, the possible reason mainly affecting the repetition rate PN is the SBL RIN. Here we calculate the theoretical transduction coefficient from soliton pump RIN to repetition rate PN. The power fluctuations of the soliton pump (here the SBL) will induce timing jitter of the repetition rate since the FSR depends on the Kerr shift related to the intra-cavity circulating optical power. Assuming a laser on resonance, the SPM induced FSR shift follows [12]

$$\begin{gathered}\frac{\delta D_1(f)}{2\pi} = \alpha \delta P(f) \\ \alpha = \frac{D_1}{2\pi}\frac{4\eta c n_2}{\kappa V_{eff} n_0^2}\end{gathered}, \tag{SE 3}$$

where $\kappa/2\pi = 0.5$ MHz is the cavity energy decay rate, $\eta = \kappa_{ext}/\kappa = 0.45$ is the coupling impedance of the resonator (κ_{ext} is the coupling rate), $V_{eff} = 5\times10^{-12}$ m^3 is the mode volume, $n_2 = 3.6\times10^{-20}$ m^2/W is the Kerr nonlinear index and $n_0 = 1.4682$ is the refractive index. These values yield a conversion coefficient $\alpha = 5.8$ kHz/W . The relationship between SBL RIN) $S_{RIN}(f)$ and the associated induced soliton phase noise $S_\phi(f)$ can be estimated by

$$S_\phi(f) = \left(\frac{\alpha}{f}P\right)^2 S_{RIN}(f). \tag{SE 4}$$

The equivalent SBL power is estimated to be $P \approx 800$ mW . The transduced repetition rate PN is displayed in Fig. 3b (blue line) in the main text, remarkably matching the measured repetition rate PN over the almost whole range of 4 kHz–1 MHz. Some difference (<6 dB) for the low offset frequencies is due to the effect of the servo in the ARMI setup and the EDFA induced SBL RIN. Therefore, we claim that the SBL RIN is the dominant factor affecting soliton timing jitter.

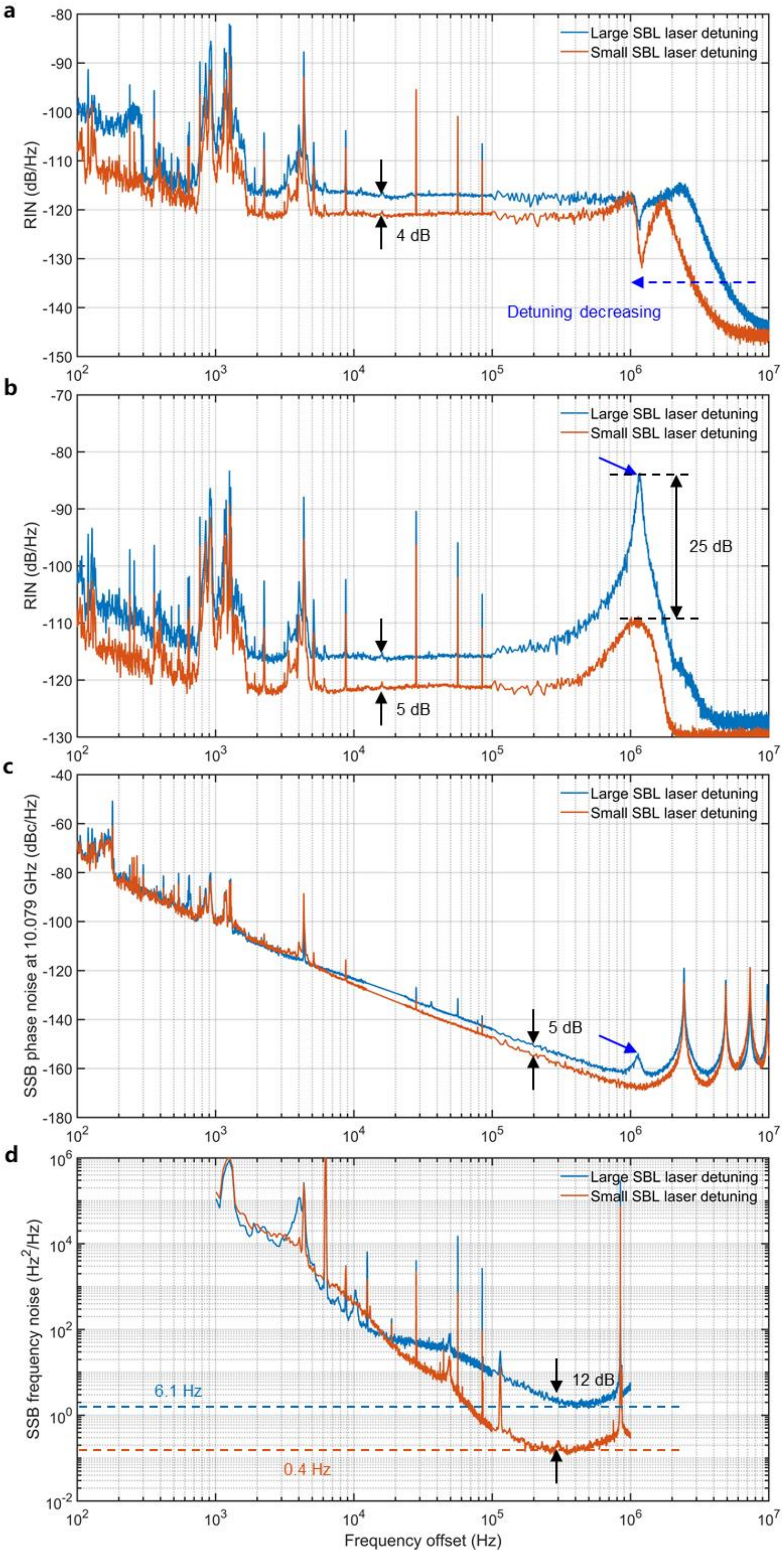

Fig. S15. Soliton performance improvement with decreased SBL laser detuning. (a) Total RIN; (b) Soliton RIN; (c) SSB phase noise of SBL soliton repetition rate; (d) SSB frequency noise of SBL.

C. Detuning noise from SBL

The detuning fluctuations will influence the stability of comb repetition rate through nonlinear effects, such as the Raman self-frequency shift and soliton recoil corresponding to dispersive wave emission [12,13]. This

PM2PM transduction process can be resulted from either soliton pump phase noise (here SBL phase noise) or cavity resonance phase noise.

However, we find that the comb repetition rate PN is not dominated by the soliton pump phase noise. There are two evidences. (i) ***SBL soliton PN follows 1/f^2 trend, while the SBL PN does not.*** According to the inset of Fig. 3a in the main text, SBL frequency noise follows pump frequency noise in the offset frequencies below 10 kHz, which is also found in other on-chip SBS microlasers [14]. At high frequencies, the SBL improves from the pump noise by 25 dB. Since the pump phase noise deviates from 1/f^2 trend (Fig. S16) by over 20 dB at low offset frequencies, the SBL phase noise should deviate from 1/f^2 trend by over (20+25)=45 dB at low offset frequencies (both the pump and SBL phase noise follows 1/f^2 trend at high offset frequencies). Assuming the transduction from SBL phase noise to the comb repetition rate phase, we can not obtain the experimental repetition rate PN roughly following 1/f^2 trend at low offset frequencies. (ii) ***The variation inconsistency between the SBL soliton PN and SBL phase noise (frequency noise).*** According to the discussion in Section B, when decreasing the SBL detuning, the SBL phase noise at high offset frequencies decreases by 12 dB while the repetition rate PN only decreases by 5 dB. The inconsistent intensity changes suggest that the soliton repetition rate PN is not determined by the SBL phase noise.

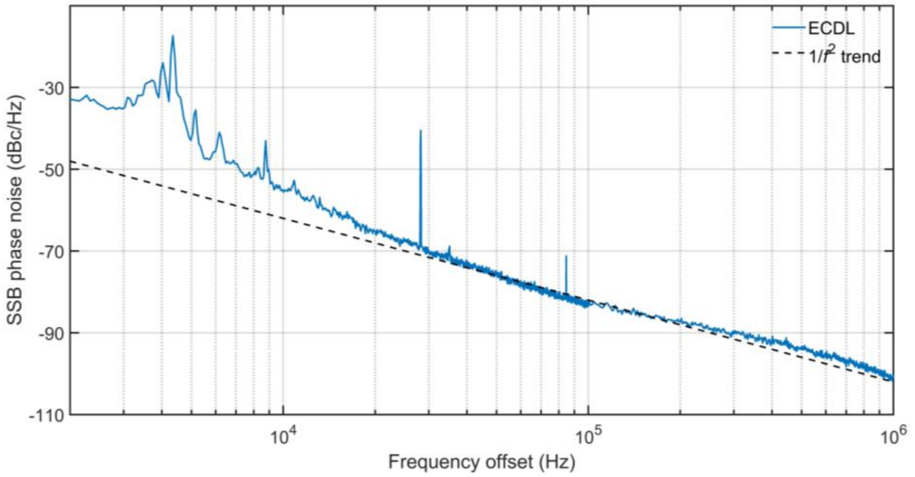

Fig. S16. SSB phase noise of ECDL by beating the ECDL with a referenced narrow-linewidth laser. The pump phase noises strongly deviate from 1/f^2 trend at low offset frequencies.

In our GRIN-MMF FP mesoresonator, nonlinear effects induced timing jitter is weak, since (i) SBL soliton spectra do not exhibit obvious dispersion wave emission; (ii) SBL soliton pulse duration is ~1 ps which sharply reduces the Raman self-frequency shift.

We will discuss the cavity resonance fluctuations due to thermal noise in Section D.

D. Resonator thermal noise limit

There are three spontaneous processes can cause the phase fluctuations of the light propagating in fibers, the thermal expansion effect, thermo-optic effect and thermal mechanical effect. Usually, the thermal-optic effect is the dominant factor for the light phase fluctuations. The fundamental thermo-dynamic fluctuations within the optical resonator mode volume result from the heat exchange between the resonator and its ambient and can lead to refractive index change through thermo-optic effect, resulting in the thermo-refractive noise (TRN) for the resonant frequencies. In general, the thermodynamic fluctuations of temperature, whose variance is given by [15]

$$\left\langle \delta T^2 \right\rangle = \frac{k_B T^2}{\rho C V}, \quad \text{(SE 5)}$$

where T is the temperature of the heat bath, k_B the Boltzmann constant, ρ the density, C the specific heat, and V the mode volume. Owing to the large mode volume of our GRIN-MMF FP mesoresonator, we can achieve notably smaller TRN than FP mesoresonator made of HNLF.

The relationship between frequency noise of the resonant frequency $S_\nu(\omega)$ and temperature fluctuations $S_{\delta T}(\omega)$ is given by [15]

$$S_\nu(\omega) = \left(\nu_0 \frac{dn/dT}{n_0} \right)^2 S_{\delta T}(\omega), \quad \text{(SE 6)}$$

where $dn/dT = 9.5\times10^{-6}\ K^{-1}$ is the thermo-optic coefficient, $n_0 = 1.4682$ is the refractive index, $\nu_0 = 193$ THz is the resonant frequency, ω is the angular Fourier frequency.

The SSB temperature noise power spectral density for a single-mode fiber is given by [16,17]

$$S_{\delta T}(\omega) = \frac{k_B T^2}{4\pi^2 \kappa_T l} \ln\left[\frac{k_{\max}^4 + \left(\frac{\omega}{D}\right)^2}{k_{\min}^4 + \left(\frac{\omega}{D}\right)^2}\right], \qquad \text{(SE 7)}$$

where $\kappa_T = 1.37\ Wm^{-1}K^{-1}$ is thermal conductivity, $k_{\max} = 2/w_0$ where $w_0 = 9$ μm is the fiber mode field radius, $k_{\min} = 2.405/a_f$ is insulating boundary conditions, where $2a_f = 125$ μm is the fiber outer diameter, $D = 0.82\times10^{-6}\ m^2/s$ is the thermal diffusivity.

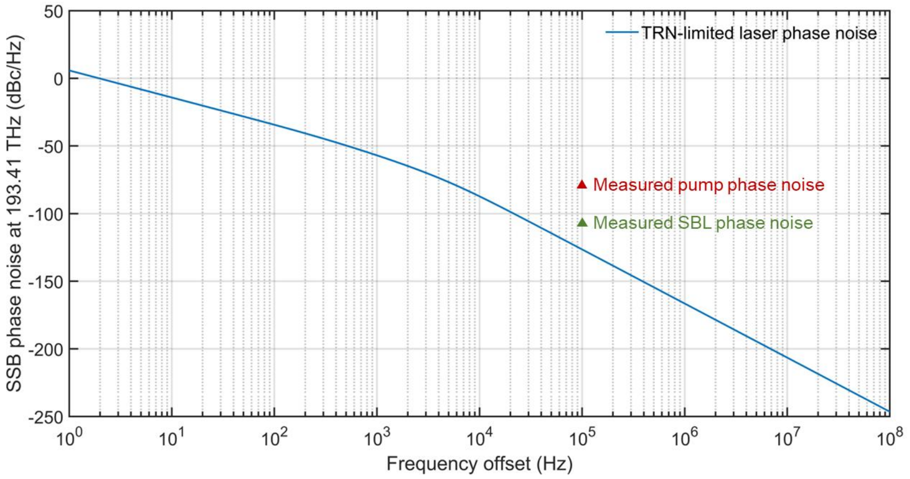

Fig. S17. TRN-limited laser phase noise in our GRIN-MMF FP mesoresonator. The measured pump phase noise (Fig. S16) as well as the SBL phase noise at 100 kHz are marked in red and green triangles.

Although Eq. (7) is designed for a single mode fiber, we can still use it to estimate the TRN since it follows the same rule by Eq. (5): increased mode volume will lead to reduced temperature fluctuations. First, we study the TRN-limited laser phase noise. Figure S17 shows the TRN-limited laser phase noise. The SBL phase noise is not limited by the thermo-refractive noise.

We then study how TRN influence the stability of comb repetition rate. The thermal fluctuations will influence the repetition rate through two channels owing to the cavity resonance fluctuation: (i) directly change the resonator FSR (minified by mode index=193 THz/10.079 GHz from cavity resonance), which is the fundamental limit imposed by TRN; (ii) detuning noise (PM2PM). In Fig. S18, we overlay the measured repetition rate PN, the quantum limit, the fundamental limit imposed by TRN, the detuning noise imposed by TRN. It can be inferred that the experimental phase noise of SBL comb repetition rate is not caused by the above-mentioned factors. Furthermore, at high offset frequencies quantum limit is thought to be the ultimate limit of the repetition rate PN in our GRIN-MMF FP mesoresonator.

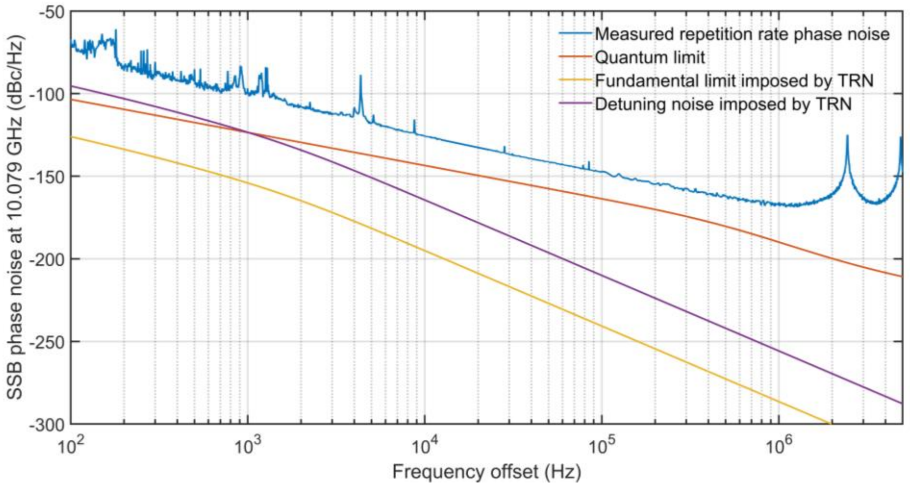

Fig. S18. Comparison of the measured repetition rate PN, quantum limit of repetition rate PN, fundamental limit of repetition rate imposed by TRN and detuning noise of repetition rate imposed by TRN. PM2PM transduction coefficient is set to be 55 dB [18], which is the value for on-chip Si_3N_4 microresonator with broad-bandwidth combs. In theory, the PM2PM transduction coefficient for our GRIN-MMF FP mesoresonators should be smaller than that in Si_3N_4 microresonators due to weak nonlinear effects, such as the Raman self-frequency shift and soliton recoil.

E. SBL RIN limit

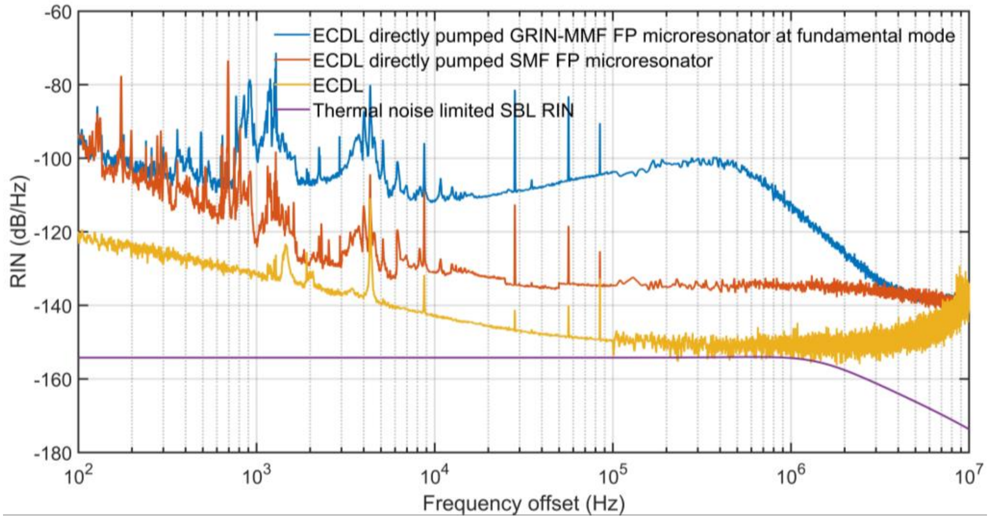

Fig. S19. RIN measurement, including output laser RIN from the GRIN-MMF FP mesoresonator, SMF FP mesoresonator and calculated thermal-noise-limited RIN for SBL without considering the cavity detuning effect.

According to the analysis above, the SBL soliton repetition rate PN is limited by the SBL RIN, which is transduced from the pump RIN and is much larger than the thermal-noise limited SBL RIN (purple line in Fig. S19, details see Appendix). Therefore, how to lower the intra-cavity pump RIN is the key to approach the quantum limit of timing jitter. In Fig. S19, we compare the output laser RIN (without any active control) from the GRIN-MMF FP mesoresonator and another step-index single-mode fiber (SMF) FP mesoresonator (cavity linewidth of ~9 MHz). Both the mesoresonators are directly pumped by the same ECDL without an amplifier. It can be clearly seen that both mesoresonators impose extra RIN to the output laser. What's more, the GRIN-MMF FP mesoresonator imposes 30 dB [~20*log(12/0.5)=27 dB] RIN larger than the SMF FP mesoresonator, which can be attributed to the difference of Q factors: RIN will be boosted much more in higher Q mesoresonators. Therefore, compromise between Q factor, pump threshold, RIN and repetition rate PN should be made. However, it is still feasible to reduce the pump RIN thus the SBL RIN by active control [14,19] in the experiment.

APPENDIX: SBL CALCULATION

This Section provides the key equations and parameters to calculate (i) the transduction coefficient from pump RIN to SBL RIN in Fig. S14; and (ii) thermal-noise-limited RIN for SBL in Fig. S19. The complete equation derivations can be found in Ref. [10]. The SBS laser parameters and their definitions along with representing symbols here follows the ones in Ref. [10].

Table S1. Simulation parameters for SBL

Parameter	Value	Parameter	Value
τ_F	$1/(2\pi\times0.5\times10^6)\,s$	ρ_0	$2200\,kg/m^3$
τ_B	$1/(2\pi\times0.5\times10^6)\,s$	S	$4.7\times10^{10}\sqrt{W/m^2F}$
τ_{ext}	$1/(2\pi\times0.4\times10^6)\,s$	Γ_b	$2\pi\times31\times10^6\,rad/s$
Λ_F	0.5	Ω_b	$2\pi\times10.343\times10^9\,rad/s$
Λ_B	0.5	Ω	$2\pi\times10.079\times10^9\,rad/s$
Λ_ρ	0.5	l_ρ/R	$1.22\times10^7\,1/m$
γ_e	1.5	V	$5\times10^{-12}\,m^3$
ω_l	$1.2153\times10^{15}\,rad/s$	V_{ph}	$5\times10^{-12}\,m^3$
n_0	1.4682		

(i) the transduction coefficient from pump RIN to SBL RIN in Fig. S14 is calculated by Eq. (30) in Ref. [10].

(ii) thermal-noise-limited RIN for SBL in Fig. S19 is calculated by Eq. (32) and Eq. (43) in Ref. [10].

Supplementary References

[1] L.G. Wright, Z.M. Ziegler, P.M. Lushnikov, Z. Zhu, M.A. Eftekhar, D.N. Christodoulides, F.W. Wise, Multimode nonlinear fiber optics: massively parallel numerical solver, tutorial, and outlook, IEEE J. Sel. Top. Quantum Electron. 24 (2017) 1–16. https://doi.org/10.1109/JSTQE.2017.2779749.

[2] A. Minardo, R. Bernini, L. Zeni, Experimental and numerical study on stimulated Brillouin scattering in a graded-index multimode fiber, Opt. Express. 22 (2014) 17480–17489. https://doi.org/10.1364/OE.22.017480.

[3] R. Suzuki, S. Fujii, A. Hori, T. Tanabe, Theoretical study on dual-comb generation and soliton trapping in a single microresonator with orthogonally polarized dual pumping, IEEE Photonics J. 11 (2018) 1–11.

[4] L.G. Wright, W.H. Renninger, D.N. Christodoulides, F.W. Wise, Spatiotemporal dynamics of multimode optical solitons, Opt. Express. 23 (2015) 3492–3506. https://doi.org/10.1364/OE.23.003492.

[5] O. Llopis, Z. Abdallah, V. Auroux, A. Fernandez, High spectral purity laser characterization with a self-heterodyne frequency discriminator, in: 2015 Jt. Conf. IEEE Int. Freq. Control Symp. Eur. Freq. Time Forum, IEEE, 2015: pp. 602–605. https://doi.org/10.1109/FCS.2015.7138917.

[6] D. Kwon, C.-G. Jeon, J. Shin, M.-S. Heo, S.E. Park, Y. Song, J. Kim, Reference-free, high-resolution measurement method of timing jitter spectra of optical frequency combs, Sci. Rep. 7 (2017) 1–9.

[7] D. Jeong, D. Kwon, I. Jeon, I.H. Do, J. Kim, H. Lee, H. Lee, H. Lee, Ultralow jitter silica microcomb, Optica. 7 (2020) 1108–1111. https://doi.org/10.1364/OPTICA.390944.

[8] S. Camatel, V. Ferrero, Narrow linewidth CW laser phase noise characterization methods for coherent transmission system applications, J. Light. Technol. 26 (2008) 3048–3055.

[9] T.J. Kippenberg, A. Schliesser, M. Gorodetsky, Phase noise measurement of external cavity diode lasers and implications for optomechanical sideband cooling of GHz mechanical modes, New J. Phys. 15 (2013) 015019. https://doi.org/10.1088/1367-2630/15/1/015019.

[10] W. Loh, S.B. Papp, S.A. Diddams, Noise and dynamics of stimulated-Brillouin-scattering microresonator lasers, Phys. Rev. A. 91 (2015) 053843. https://doi.org/10.1103/PhysRevA.91.053843.

[11] Z. Yuan, H. Wang, L. Wu, M. Gao, K. Vahala, Linewidth enhancement factor in a microcavity Brillouin laser, Optica. 7 (2020) 1150–1153.

[12] E. Lucas, P. Brochard, R. Bouchand, S. Schilt, T. Südmeyer, T.J. Kippenberg, Ultralow-noise photonic microwave synthesis using a soliton microcomb-based transfer oscillator, Nat. Commun. 11 (2020) 1–8. https://doi.org/10.1038/s41467-019-14059-4.

[13] Q.-F. Yang, Q.-X. Ji, L. Wu, B. Shen, H. Wang, C. Bao, Z. Yuan, K. Vahala, Dispersive-wave induced noise limits in miniature soliton microwave sources, Nat. Commun. 12 (2021) 1–10.

[14] W. Loh, A.A. Green, F.N. Baynes, D.C. Cole, F.J. Quinlan, H. Lee, K.J. Vahala, S.B. Papp, S.A. Diddams, Dual-microcavity narrow-linewidth Brillouin laser, Optica. 2 (2015) 225–232.
[15] G. Huang, E. Lucas, J. Liu, A.S. Raja, G. Lihachev, M.L. Gorodetsky, N.J. Engelsen, T.J. Kippenberg, Thermorefractive noise in silicon-nitride microresonators, Phys. Rev. A. 99 (2019) 061801.
[16] K.H. Wanser, Fundamental phase noise limit in optical fibres due to temperature fluctuations, Electron. Lett. 28 (1992) 53. https://doi.org/10.1049/el:19920033.
[17] L. Duan, General treatment of the thermal noises in optical fibers, Phys. Rev. A. 86 (2012) 023817. https://doi.org/10.1103/PhysRevA.86.023817.
[18] J. Liu, E. Lucas, A.S. Raja, J. He, J. Riemensberger, R.N. Wang, M. Karpov, H. Guo, R. Bouchand, T.J. Kippenberg, Photonic microwave generation in the X- and K-band using integrated soliton microcombs, Nat. Photonics. (2020) 1–6. https://doi.org/10.1038/s41566-020-0617-x.
[19] W. Loh, J. Becker, D.C. Cole, A. Coillet, F.N. Baynes, S.B. Papp, S.A. Diddams, A microrod-resonator Brillouin laser with 240 Hz absolute linewidth, New J. Phys. 18 (2016) 045001. https://doi.org/10.1088/1367-2630/18/4/045001.
